# Pathways for Genome Integrity in G2 Phase of the Cell Cycle

**DOI:** 10.3390/biom2040579

**Published:** 2012-11-30

**Authors:** Arne Nedergaard Kousholt, Tobias Menzel, Claus Storgaard Sørensen

**Affiliations:** Biotech Research and Innovation Centre, University of Copenhagen, Ole Maaløes Vej 5, DK-2200 Copenhagen N, Denmark, ank@bric.ku.dk (A.N.K.); TME@bric.ku.dk (T.M.)

**Keywords:** Genome integrity, IR, DNA damage checkpoint, G2/M transition, DNA repair

## Abstract

The maintenance of genome integrity is important for normal cellular functions, organism development and the prevention of diseases, such as cancer. Cellular pathways respond immediately to DNA breaks leading to the initiation of a multi-facetted DNA damage response, which leads to DNA repair and cell cycle arrest. Cell cycle checkpoints provide the cell time to complete replication and repair the DNA damage before it can continue to the next cell cycle phase. The G2/M checkpoint plays an especially important role in ensuring the propagation of error-free copies of the genome to each daughter cell. Here, we review recent progress in our understanding of DNA repair and checkpoint pathways in late S and G2 phases. This review will first describe the current understanding of normal cell cycle progression through G2 phase to mitosis. It will also discuss the DNA damage response including cell cycle checkpoint control and DNA double-strand break repair. Finally, we discuss the emerging concept that DNA repair pathways play a major role in the G2/M checkpoint pathway thereby blocking cell division as long as DNA lesions are present.

## 1. Introduction

It is essential for the survival and function of living cells to safeguard genomic integrity and to ensure the proper transmission of genetic information encoded by DNA. This requires precision during DNA replication, the proper partitioning of chromosomes to daughter cells during cell division and the ability to identify and correct DNA lesions that arise spontaneously or are induced by exogenous agents. The accumulation of these mutations and the resulting genetic instability can promote aging, genetic diseases and oncogenesis. In the latter case by activating cellular oncogenes and inactivating tumor suppressor genes [1,2,3].

In this review, we present major pathways that govern genome integrity in the G2 phase of the cell cycle. We highlight the strong interplay between the control of the unperturbed cell cycle transition, and the DNA damage signaling initiated by various DNA damage structures. In particular, we focus on how the DNA Double Strand Break (DSB) activates the checkpoint and initiation of repair. Furthermore, we provide an overview of the different phases of the checkpoint, from initiation and maintenance to recovery. For each phase, different pathways play a more critical role in the control. This is supported by recent findings from our and other labs, which shed new light on how repair and checkpoint control are coordinated [4,5,6].

## 2. Regulation of Cell Cycle Progression through G2 Phase

Faithful transmission of genetic information in cellular organisms is carried out by two basic processes: DNA replication and cell division. During the S-phase, DNA synthesis takes place to completely replicate the double stranded DNA molecule. Two gap phases precede the S and M phases; in G1 the cell prepares for DNA synthesis, and in G2 the cell prepares for the mitotic division that takes place in the M phase. Together, the G1, S, and G2 phases are referred to as interphase. After completing DNA synthesis and progression through the G2 phase, the cell divides in mitosis by segregating the chromosomes into two separate daughter cells. Stages of mitosis include prophase, metaphase, anaphase and telophase [7]. 

### 2.1. Regulation of the Cell Cycle via Cyclin-Dependent Kinases

The key cell cycle drivers are the Cyclin-dependent kinases (CDK), a family of serine/threonine kinases. Different CDKs play a role at different steps of the cell cycle: CDK2, 4, and 6 are active during G1, CDK2 during G1 and S phase and CDK1 during G2 and mitosis [8,9,10]. CDK activity is controlled by several mechanisms, which include the accumulation of the activating subunit Cyclin, the subcellular localization, CDK phosphorylation status, and CDK inhibitors (CKI). In order to coordinate the progress of the cell cycle via CDK activity, positive feedback loops build up throughout the cell cycle; once a certain threshold has been reached, the cell commits to the next cell cycle phase. In this review, we will focus on the control of CDK activity for the G2/M transition.

The first level of CDK activity regulation is the presence of a binding partner, the Cyclins. Whereas CDK protein levels remain stable, Cyclin levels ﬂuctuate throughout the cell cycle, thereby regulating CDK activity. Different Cyclins bind to different CDKs to control the cell cycle at different steps: D-type Cyclins associate with CDK4 and 6, regulating G1 progression. CDK2 can bind Cyclin E to control S phase entry or Cyclin A during the S phase, and CDK1-Cyclin A binding promotes cell cycle progression during the G2 phase. The Cyclin B-CDK1 complex, also referred to as the mitosis-promoting factor (MPF), plays an important role for mitotic entry and during mitosis [8,9,10]. Cyclin B level is high in G2 and early mitosis, and it is regulated via periodical transcription and protein degradation cycles throughout the cell cycle. Transcription of Cyclin B is under the control of the transcription factors NF-Y, FoxM1 and B-Myb, which are all activated by Cyclin A-CDK1 activity in S and G2. Thus, Cyclin B transcription starts in the S phase, peaking in late G2 [11,12,13]. The degradation of Cyclin B is regulated by the anaphase-promoting complex/cyclosome (APC/C) [14], an E3 ubiquitin ligase that targets specific proteins for proteasomal degradation. Cyclin B polyubiquitylation starts in metaphase, where it is critical for mitotic exit, and it continues until the early S phase where the polyubiquitylation is downregulated due to APC/C phosphorylation by Cyclin-CDK2 and binding of the APC/C inhibitor Emi1 [15,16].

Another important aspect of CDK regulation is cellular localization. Most CDK1-Cyclin B substrates that play a role during mitosis are nuclear proteins, indicating that at least a subset of the Cyclin B-CDK1 complex needs to be in the nucleus to promote mitosis [17,18]. In the interphase, Cyclin B shuttles between the nucleus and the cytoplasm; it is actively exported from the nucleus by the export protein CRM1 during the S phase and the major part of G2, counteracting a constitutive import into the nucleus by Importinβ [19,20]. The CRM1 binding site of Cyclin B is phosphorylated in late G2 by CDK1 and PLK1, inhibiting CRM1-mediated export. This results in a Cyclin B inﬂux into the nucleus, where Cyclin B can bind and activate CDK1 [21]. Even though the role and timing of PLK1-mediated phosphorylation of Cyclin B is still debated, there is evidence that a feedback loop is formed where CDK1 enhances its own activation by promoting the nuclear import of Cyclin B. Indeed, recent experiments using a probe that specifically measures Cyclin B-CDK1 activity in living cells has shown that Cyclin B-CDK1 is rapidly localized to the nucleus following its initial activation in the cytoplasm [22]. In addition, a large fraction of Cyclin B accumulates at the centrosomes in late G2. This facilitates interactions between Cyclin B and other cell cycle regulators such as PLK1, AURORA A, CDC25B and C, and may be the initial site of Cyclin B phosphorylation. [23]. 

Adding to the complex regulation of CDK activity is the phosphorylation status of CDKs. CDK1 activity can be directly modulated by activating and inactivating phosphorylation events [7]. The CDK activating kinase (CAK), consisting of the Cyclin H-CDK7 complex, phosphorylates CDK1 on T161 within its T loop, mediating its activation. The inactivating phosphorylations of CDK1 on T14 and Y15 by the kinases WEE1 and MYT1 can be removed by CDC25 phosphatases [17,18]. A final major level of CDK control consists of the CDK inhibitors (CKI). Two CKI families have been identified, the INK4 family CKIs compete with Cyclins for CDK binding and specifically inactivate G1 CDKs. The CIP/KIP family of CKIs inactivates Cyclin-CDK complexes during G1 and also Cyclin B-CDK1 complexes at the G2/M transition [24].

### 2.2. Regulation of CDK1 Activity at the G2/M Transition

Once activated, the Cyclin B-CDK1 complex can phosphorylate a number of substrates required for mitotic entry and cytokinesis, e.g., generation of the mitotic spindle, chromosome condensation and nuclear envelope breakdown [25]. Control of Cyclin B-CDK1 activity may take place at two levels; a direct control of Cyclin B-CDK1 phosphorylation status by WEE1, MYT1, and CDC25, as well as a more indirect regulation via the AURORA A /BORA/PLK1 network [18].

#### 2.2.1. Control of CDK1-Cyclin B via Key Phosphorylation Sites

The catalytic activity of CDK1 is inhibited during the S and G2 phases due to phosphorylation on T14 and Y15 by the kinases WEE1 and MYT1, and these phosphorylations are removed by CDC25 phosphatases to mediate kinase activation [26,27]. Thus, WEE1/MYT1 and CDC25 activity constitute a switch for turning on and off CDK1 activity. Once Cyclin B-CDK1 is active, it phosphorylates and inactivates WEE1 and MYT1 by initiating WEE1 degradation and MYT1 inhibition, creating a positive feedback loop in CDK1 activation [28,29]. Only low levels of CDK1 activity can be detected already in the G2 phase, whereas CDK1 activity peaks in the last 30 minutes preceding the prometaphase [30]. In the G2 phase, active WEE1 is in the nucleus where it continuously inactivates Cyclin B-CDK1. The inactive Cyclin B-CDK1 cannot maintain its phosphorylation on T161 in the T loop. After export to the cytoplasm, CDK1 can be reactivated and it autophosphorylates in the T loop again, increasing its nuclear import via Cyclin B phosphorylation. Thus, Cyclin B-CDK1 activity needs to overcome a threshold to promote CDC25 activation and WEE1 inactivation in order to globally activate Cyclin B-CDK1 activity [18,31]. 

Three CDC25 isoforms exist, termed CDC25A, CDC25B and CDC25C. They differ in terms of regulation and importance for the cell cycle progression, but they appear to cooperate in regulating the G2/M transition [32]. All CDC25 isoforms can remove the inhibitory phosphorylations on T14 and Y15 of CDK1 and thereby promote mitotic entry. Active CDK1 stabilizes CDC25A, prevents nuclear export of CDC25B and activates CDC25C [33,34,35]. Thus, by activating CDC25 phosphatases and at the same time by inhibiting WEE1 and MYT1 kinases, CDK1 amplifies its own activation [26]. At the end of mitosis, CDC25A and B are degraded by ubiquitin-mediated proteolysis [32]. All CDC25 isoforms shuttle between the nucleus and the cytoplasm in the G2 phase, and CDC25A appears to be predominantly nuclear. However, the importance of CDC25 subcellular localization remains unclear. CDC25B is also located to the centrosomes in the G2 phase where it is suggested to be involved in initiating the activation of Cyclin B-CDK1 [36].

#### 2.2.2. CDK1-Cyclin B Regulation by the AURORA A/BORA/PLK1 Pathway

In addition to the direct regulation of CDK1 by Cyclin B binding and by its phosphorylation status, several feedback mechanisms around the AURORA A/BORA/PLK1 network indirectly regulate CDK1 activity. The AURORA A kinase is located at the centrosome to coordinate centrosome maturation, spindle assembly, and asymmetric protein localization during mitosis [37]. AURORA A can phosphorylate the polo-like kinase PLK1, at the G2/M transition [38,39]. Moreover, AURORA A protein levels correlate with PLK1 levels: They rise in G2 and peak in mitosis until AURORA A and PLK1 are degraded in an APC/C-mediated manner at mitotic exit [37]. The kinase activity of AURORA A requires autophosphorylation of T288 in its activatory T loop, which is facilitated by cofactors such as TPX2, AJUBA, and BORA [40,41,42,43,44]. PLK1 does not only work downstream of AURORA A, it also targets AURORA A to the centrosome and spindle poles. This process requires PLK1-mediated degradation of BORA during early mitosis [45,46]. Additionally, AURORA A phosphorylates CDC25B and can hence stimulate Cyclin B-CDK1 independent from PLK1 [47]. BORA may play a particularly important role in the activation of PLK1 by AURORA A. BORA binds AURORA A [42] as well as PLK1 [39,45], and it enhances the ability of AURORA A to directly phosphorylate PLK1 on T210 [39]. This may be especially relevant during checkpoint recovery [38], and depletion of BORA or AURORA A leads to a delay in mitotic entry and blocks G2/M checkpoint recovery. 

Besides its role during mitosis, PLK1 is a regulator of the G2/M transition, in particular in response to DNA damage. PLK1 modulates regulators of Cyclin B-CDK1, such as WEE1, MYT1, and CDC25C, to finally promote mitotic entry. CDK1 phosphorylates its inhibitory kinase WEE1, targeting it for a second phosphorylation by PLK1. In addition, PLK1 can activate Forkhead Box M1 (FoxM1) transcription factor and thereby enhance the transcription of several mitotic entry genes, like Cyclin B, CDC25B, and PLK1 itself [48,49]. Importantly, depletion of single mitotic entry regulators has no or only little effect on mitotic entry. Instead, it can lead to abnormal cell division and mitotic abnormalities, indicating that many factors are largely redundant for mitotic entry but essential for proper orchestration of mitotic events [18]. For instance, undamaged PLK1-depleted cells enter mitosis with high levels of WEE1, indicating that the effect of PLK1 on WEE1 is not essential for mitotic entry in unperturbed cells. However, the AURORA A/BORA/PLK1 network plays an essential role in preventing adaptation, the mitotic entry of cells with persistent or irreparable DNA damage [38,50].

## 3. Cellular Responses to DNA DSBs

The accumulation of mutations and the resulting genomic instability lie at the origin of developmental diseases and cancer [2]. To maintain genomic integrity, cells have developed a complex network of mechanisms in order to detect, signal and repair DNA damage. This network is termed the DNA damage response (DDR). Once the DNA lesions are detected, cellular pathways can promote a number of outcomes depending on the severity of the damage and the characteristic of the specific cell lineage. Possible outcomes include slowing down or blocking the cell cycle progression, allowing time for repair of the lesions or initiation of an apoptotic program. In either case, the aim is to prevent the damaged DNA from being replicated or inherited into the daughter cells [51].

DNA DSBs are very deleterious DNA lesions, which can result from exogenous agents such as ionizing radiation (IR) and chemotherapeutic drugs. Also, they can arise due to endogenous processes such as the production of reactive oxygen intermediates or the collapse of stalled DNA replication forks [1,2]. In addition, DSBs are generated physiologically to facilitate important developmental processes such as meiosis, as well as V(D)J and Class Switch Recombination that are essential for proper immune system development [52].

In the following section we will focus on the response to IR-induced DNA DSBs, since these are lesions that dividing cells must deal with prior to mitotic entry to avoid uneven division of the genome. In line with this, IR-induced DNA lesions lead to strong activation of the G2/M checkpoint. Other types of DNA lesions, such as single-base changes can be dealt with in the following cell cycle and do not readily activate the checkpoint [53]. Interestingly, a study has found that a certain number of DNA DSBs is needed for checkpoint activation [54]. This suggests that proliferation of cells may be more beneficial for the organism than tight checkpoint control. Moreover, it is possible that the remaining DNA lesions are repaired during the following G1 phase, thereby counteracting genomic instability [55].

### 3.1. Repair of DNA DSBs

DNA DSBs represent very dangerous lesions, and at least four independent DSB repair pathways have evolved to protect the genome; homologous recombination (HR), two mechanistically different forms of end joining-classical and alternative non‐homologous end joining (c‐NHEJ and alt‐NHEJ), and single‐strand annealing (SSA). Alt-NHEJ is also referred to as microhomology‐mediated end‐joining (MMEJ) [1,56,57]. Major determinants for the choice of DSB repair pathway are the extent of DNA end processing and cell cycle position. The induction of a DNA DSB can give rise to a variety of chemically heterogeneous ends, including those with modified terminal nucleotides or even protein-DNA adducts, referred to as ‘dirty’ ends. These ends require processing by nucleases or other DNA modifying enzymes to enable repair by HR or NHEJ [58]. This is called DNA end processing and can include nucleases removing a few nucleotides from one or more of the strands. On the other hand, DNA end resection refers to the removal of up to kilobases of nucleotides from the 5’ strand, leading to long RPA coated 3’ ssDNA strand overhangs. 

DNA end resection is required to initiate HR, SSA, and alt‐NHEJ. On the contrary, c‐NHEJ does not require resection of the DNA break for repair initiation. For alt-NHEJ, DSB resection is limited (5‐25 nt), and more extensively so for HR and SSA [59,60]. Another determinant for the choice of repair pathway is the cell cycle phase. HR-mediated repair is generally limited to the S and G2 phase where a sequence identical sister chromatid is available. Importantly, HR seems to be used for more specialized DSBs, since even in S and G2 it has been estimated that only around 15% of all DSB are repaired by HR [61]. Therefore, the majority of DSBs occurring throughout the cell cycle are repaired by NHEJ. It should be noted that DSBs are repaired rapidly by NHEJ, whereas the process of HR-mediated repair can take several hours. Moreover, a recent study suggests that HR is predominantly utilized for repair of DSBs in areas of heterochromatin [61,62,63].

#### 3.1.1. Homologous Recombination

HR is involved in the repair of DSBs as well as DNA lesions that occur at replication forks. During HR repair, an intact homologous sequence is used as a template for accurate DSB repair. It is of great importance to keep the broken DNA ends, as well as the sister chromatids, in close proximity once the DSB has occurred. In mammalian cells, this is accomplished by the MRN and cohesin complexes, including members of the structural maintenance of chromosomes (SMC) family of proteins. The MRN complex consists of MRE11 (meiotic recombination 11), RAD50, and NBS1 (Nijmegen breakage syndrome 1 protein). It plays a major role in the DNA damage response and will be described further in the following section [64,65,66]. The broken dsDNA ends are then resected in a process mediated by CtIP and MRN, leading to 3’ ssDNA overhangs of up to kilobases in length, and the recruitment of RPA (Replication Protein A) to the ssDNA [67]. 

The exact mechanism behind DNA end resection is still under investigation, but model organisms such as the yeast, *S. cerevisae*, have provided significant insights. This seminal work has led to a 2-step model of DNA resection that is initiated by the MRX (Mre11/Rad50/Xrs2) complex and the CtIP orthologue, Sae2 [58]. Following initiation, more extensive ssDNA is created through the action of the Sgs1 helicase and the Dna2 and Exo1 exonucleases [68]. Existing biochemical and cellular data suggest that the mammalian orthologues carry out similar roles with MRN and CtIP initiating resection, and that the BLM helicase and EXO1 and DNA2 exonucleases carry out the subsequent, more processive step of resection [67,69,70]. 

This proposed, unidirectional model of resection requires nucleases with a 5’-3’ polarity, such as Exo1. As the exonuclease activity of Mre11 is 3’-5’, it remains unclear precisely how MRN would initiate resection and what the actual nuclease activity is that catalyzes this step [71]. In a recent study in yeast, the use of an Mre11 separation of function mutation suggests that Mre11 may utilize its endonuclease activity to make a nick near the break and catalyze resection of up to 300 bp in the 3’-5’ direction [72]. Based on these findings, a bidirectional model of DNA resection has been proposed where the Mre11 3’-5’ exonuclease activity and the 5’-3’ exonuclease activities of Exo1 or Sgs1-Dna2 would catalyze resection in opposite directions of the same strand. Further experiments are needed to test this model in human cells and elucidate the details and regulation of resection [73].

Extended DNA end resection is observed in human cells following the induction of a DNA DSB, and the heterotrimeric complex RPA (subunits RPA1, RPA2, RPA3) stabilizes ssDNA [74,75]. Following the loading of RPA, BRCA2, together with PALB2 and BRCA1, loads RAD51 onto the ssDNA, displacing RPA and forming a RAD51-ssDNA nucleoprotein filament which can invade the homologous dsDNA template [76,77,78,79,80]. DNA synthesis at the damaged DNA strand is accomplished by DNA polymerases and their accessory factors, using the undamaged sister chromatid as a template [81]. Upon completion of repair, the sister chromatids are separated again. Importantly, the RPA coated ssDNA has an important role in ATR‐mediated checkpoint activation that will be described later. 

#### 3.1.2. Non-Homologous End-Joining

Non-homologous end-joining (NHEJ) is the predominant DNA DSB repair pathway in mammalian cells and it also constitutes a major DSB repair pathway in the G2 phase. During NHEJ, the broken DNA ends are rejoined directly, largely independent from DNA homologies, allowing for NHEJ to occur throughout the cell cycle [82]. In the initial step of NHEJ, the broken DNA ends are bound by the Ku70/Ku80 heterodimer within seconds; this protects them from degradation and recruits DNA-PKcs to form the DNA-PK holoenzyme [83,84]. The serine/threonine kinase activity of DNA-PKcs is greatly increased by the presence of both dsDNA ends and Ku [85]. DNA-PKcs is thought to play a critical role in preventing DNA end resection by a series of autophosphorylation events on two well characterized autophosphorylation clusters termed the ABCDE and PQR clusters [86]. Following DNA DSB binding, DNA-PKcs autophosphorylation on the ABCDE cluster results in destabilization of the DNA-PKcs interaction with the DNA ends, thus providing access for end processing enzymes [86]. Excessive end processing is then prevented by DNA-PKcs autophosphorylation on the PQR cluster, which helps protect the DNA ends [86]. Many NHEJ factors have been identified as targets of DNA-PKcs kinase activity, but little evidence that these phosphorylation events are critical for NHEJ *in vivo* has been provided [87,88,89]. On the contrary, DNA-PKcs autophosphorylation appears to be critical as mutations in the ABCDE cluster lead to radiosensitivity and V(D)J recombination defects [90,91].

Following the initial recruitment of DNA-PKcs, Artemis is recruited to resected ssDNA overhangs to create blunt DNA ends, which subsequently are religated by the XRCC4/LigaseIV complex [92,93]. Compared to HR, NHEJ does not have the capacity to repair DSBs accurately if there is loss of sequence information at the junctions. Complex DSBs require some degree of DNA end processing during NHEJ, which can lead to the elimination of DNA bases. Moreover, translocations can occur if two DNA ends from different chromosomes are ligated during NHEJ [94]. 

#### 3.1.3. Alternative Non‐homologous End-Joining

The alt-NHEJ DSB repair mechanism functions independently of Ku- and LigaseIV. Since distal microhomologies are frequently used, this pathway is also referred to as microhomology‐mediated end-joining (MMEJ). Importantly, the use of microhomologous sequences during the alignment of broken DNA ends results in deletion mutations at repair junctions. It is still debated whether alt‐NHEJ plays an important role in the repair of a wide range of DSBs, functions as a backup pathway for the classical NHEJ pathway (c-NHEJ), or primarily repairs more specialized types of DSB, for example during the process of class switch recombination CSR [95,96,97].

In order to expose microhomologies in close proximity of a DSB, nucleolytic DNA end resection is required [98]. In yeast, several factors have been implicated, including the orthologues of human MRE11, EXO1 and CtIP [98,99]. CDK activity is required for DNA end resection activity and is therefore in principle restricted to the S/G2 phases of the cell cycle. However, it has been observed that limited end resection, needed for MMEJ, can occur in G1 cells [100,101]. In addition, the activity of DNA helicases might provide an alternative mechanism to expose microhomologous sequences located adjacent to the DSB. Before ligation, the exposed microhomology is subsequently annealed and the remaining, non‐complementary 3’ flaps are removed. For this process, the structure-specific endonuclease XPF‐ERCC1 has a key role [102]. It is possible that error‐prone polymerases might act in the processing steps before repair is completed, since inserted nucleotides are observed at the MMEJ junctions. Finally, in mammalian cells, alt‐NHEJ is completed through the action of DNA ligase I or DNA ligase III, which act in concert with XRCC1 [103,104]. 

#### 3.1.4. Regulation of DSB Repair

The repair of DNA DSBs is a highly regulated process that is influenced by the structure of DNA ends and the phase of the cell cycle in which the break occurs. Both MRN and Ku protein complexes can recognize DNA ends, but they initiate different modes of repair. Importantly, cells lacking NHEJ genes reveal a DNA DSB repair bias in favor of HR, suggesting that the two repair pathways compete with each other [105]. In support of this notion, the MRN complex has the ability to displace Ku from DNA ends in order to facilitate HR [106,107]. On the contrary, Ku has been shown to protect the DNA ends from Exo1 mediated DNA end resection [108,109]. Interestingly, a recent study has suggested that Ku rapidly binds the broken ends, and it is only if repair cannot proceed with normal kinetics that Ku is displaced and the HR pathway takes over [63]. These and other studies imply that the different repair pathways may not simply compete, but can act sequentially for DNA DSB repair [110].

Another layer of regulation of the repair pathway choice comes from the control of protein levels and post-translational modifications (PTMs) throughout the cell cycle for key HR proteins. As an example, protein levels for CtIP are low during G1 and peak during the S and G2 phases [111]. A number of proteins involved in HR require phosphorylation or binding by CDK for full function, e.g., NBS1, MRE11 and CtIP. Additional PTMs found to be important for the regulation of DSB repair include sumoylation and ubiquitylation. Several E3 ubiquitin ligases and ubiquitin binding proteins have been found to be involved, directly or indirectly, in the repair of DNA DSBs [112]. One of the proteins, BRCA1, has been suggested to play a critical role in the pathway choice between HR and NHEJ. Mice expressing an alternatively spliced variant of BRCA1 that lacks exon 11 (i.e., inactive protein) harbor increased chromosomal instability and tumorigenesis. However, the ubiquitin ligase activity does not seem to be essential for these functions [113]. Interestingly, the chromosomal abnormalities and defects in HR associated with loss of BRCA1 are rescued by 53BP1 depletion [114,115]. Therefore, it appears that 53BP1 and BRCA1 are important factors in mediating DSB repair pathway choice.

### 3.2. Detection and Integration of the DNA DSB Signal

Cells have developed a complex network of mechanisms that enable them to detect the DNA damage, amplify the signal, and orchestrate damage repair and checkpoint response. In the following section, we will present these important pathways.

#### 3.2.1. Detecting the DSB - the MRN Complex

The MRN complex has been suggested to be the initial detector of DSBs. MRE11 forms the core of the MRN complex that binds DNA via its RAD50 subunit and thereby bridges the two dsDNA ends, leading to a stabilization of the DSB [73,116,117,118]. NBS1 rapidly assembles at DSB sites as seen in live human cells [119]. The initial assembly of MRN to DSBs is independent of MDC1 (mediator of DNA-damage checkpoint 1) and γH2AX, two early acting mediators of the DNA damage signal, making the MRN complex a plausible DSB detector [119,120].

#### 3.2.2. Activation of ATM and Its Downstream Targets

ATM (Ataxia Telangiectasia Mutated) is the gene mutated in the human ataxia telangiectasia (AT) syndrome, an inherited genetic instability syndrome. Cells derived from AT patients exhibit hypersensitivity to ionizing irradiation, increased chromosomal breakage, and cell-cycle checkpoint defects, indicating that ATM is important for initiating checkpoint arrest and repair after DNA damage [121,122,123]. The ATM gene encodes a 370 kDa protein that is a member of the PIKK (phosphatidylinositol 3-kinase related kinases) family of serine-threonine protein kinases. Other mammalian PIKK members are ATR (ATM- and Rad3-related) and DNA-PKcs (DNA-dependent protein kinase catalytic subunit). 

**Figure 1:**
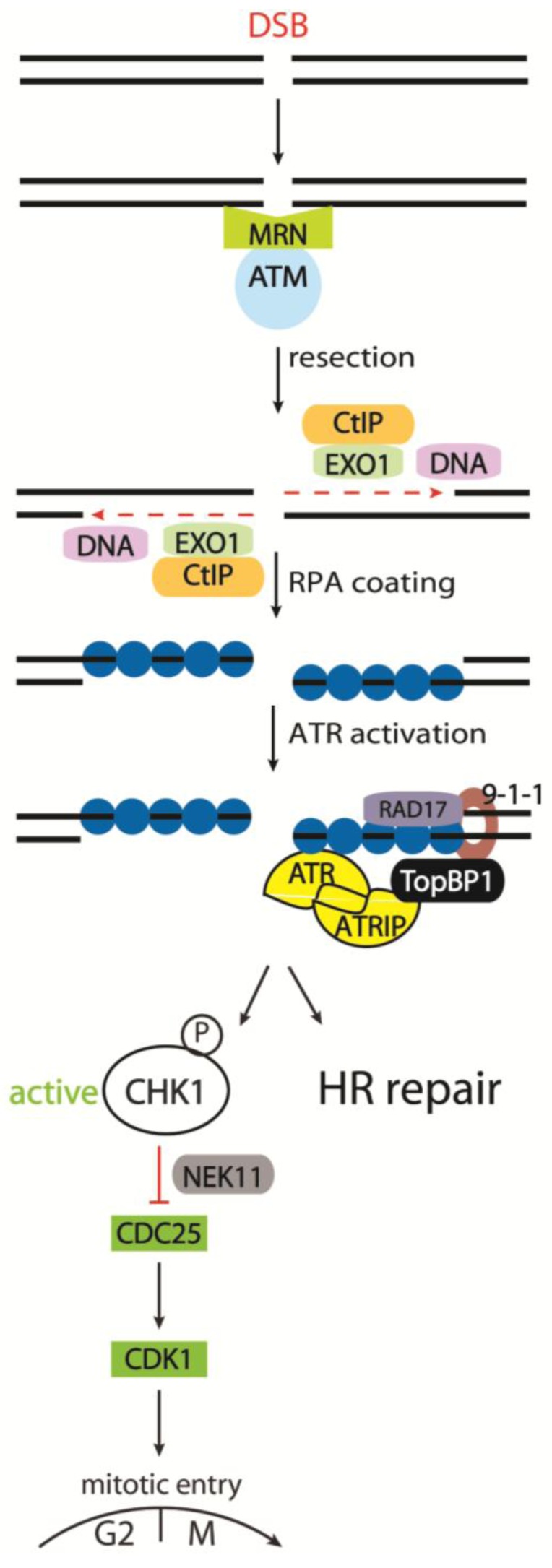
Activation of checkpoint regulator (CHK1) in response to DNA Double Strand Breaks (DSBs). The MRN complex detects DSBs and recruits Ataxia Telangiectasia Mutated (ATM) to initiate checkpoint signaling. During the S and G2 phases of the cell cycle, dsDNA resection can be performed by the nucleases DNA2 and EXO1, together with CtIP. The resulting ssDNA is coated by Replication Protein A (RPA), and is prepared for homologous recombination (HR) repair. Furthermore, ssDNA-coated by RPA recruits the ATR/ ATR interacting protein (ATRIP) complex as well as TopBP1 via the 9-1-1 complex, enabling full ATR activity. ATR then activates a subset of targets, including CHK1. CHK1 can phosphorylate CDC25A in response to DSBs, targeting CDC25A for proteasomal degradation. Since CDC25A is required for CDK1 activity to facilitate mitotic entry, cells arrest in the G2 phase upon CDC25A degradation.

The ATM kinase is an important player in DNA damage responses. In unperturbed cells, ATM is thought to be sequestered as a dimer or a higher-order multimer, masking and inactivating its kinase domain. ATM may be recruited to DSB sites through binding to the C-terminus of NBS1 [124,125]. The activation process likely involves ATM autophosphorylation on S1981, as well as on S367 and S1893 [126,127]. The ATM dimers then dissociate, unmasking the kinase domain, thus activating ATM. It is still not clear if the recruited ATM is already active or activated by the recruitment step [125,126,128,129]. However, recent data suggests the involvement of chromatin via the acetyltransferase TIP60. MRN targets TIP60 together with ATM to DNA where TIP60 interacts with trimethylated histone H3 to acetylate ATM and thereby promote ATM kinase activity [130,131]. The activated ATM can then phosphorylate several DDR proteins, including NBS1 to further amplify ATM activation [132,133]. Among other ATM phosphorylation targets are several proteins involved in the DNA DSB response including the signaling proteins H2AX and MDC1, DNA damage repair factors (CtIP, BRCA1, RAD51 and FANCD2), checkpoint regulators (CHK2, p53, MDM2) as well as KAP1 (Krüppel-associated box (KRAB) associated protein 1), a protein that is involved in global chromatin compaction [134,135]. The phosphorylation of KAP1 by ATM promotes heterochromatin relaxation, which facilitates DSB repair or processing in otherwise inaccessible heterochromatic regions [136]. Moreover, ATM, together with CtIP and MRN, initiates DSB resection and single stranded DNA (ssDNA) formation, a prerequisite for homologous recombination (HR) repair and ATR-CHK1-dependent signaling during S and G2 [67,137] (See Figure 1). 

#### 3.2.3. The Role of Phosphorylated Histone H2AX

One of the ATM targets is the histone H2AX, which can be phosphorylated by ATM [138] but also by other PIKK kinases, such as ATR (in response to replicative stress) and DNA-PKcs [139,140]. Several thousand phosphorylated H2AX (γH2AX) molecules, located around the DNA DSB, form microscopically visible nuclear foci within only minutes after ionizing radiation and can thus be used as a marker for DSBs. The number of foci increases within the first 10-30 minutes following IR, and then gradually decreases due to the elimination and repair of the DSBs [141]. γH2AX foci may function as a platform to recruit DDR factors like the MRN complex, which can then further recruit and activate additional ATM molecules, leading to an amplification of the initial signal. Strikingly, γH2AX accumulates repair and signaling factors to chromatin regions distal to DSBs, following their initial (γH2AX independent) migration to DSBs. However H2AX deficiency only has subtle effects on cell cycle checkpoints and DNA repair, indicating that it might regulate repair of selected DSBs or assist specific repair pathways, such as support 53BP1-mediated suppression of DNA end resection [62,142,143,144]. In line with this, H2AX phosphorylation has been proposed to regulate repair pathway choice, promoting HR in favor of SSA [145].

#### 3.2.4. The Molecular Pathways Controlled by ATR and DNA-PKcs

Most ATM targets are still phosphorylated to a certain degree in ATM-deficient AT-cells following ionizing radiation (IR), suggesting that other PIKK proteins are involved in the cellular response to IR. DNA-PKcs, described earlier in the NHEJ section, mainly responds to DSBs and plays a role in repairing DSBs via NHEJ. In addition, DNA-PKcs phosphorylates H2AX and has been suggested to function in DNA damage signaling, especially in promoting apoptosis and activation of the G2/M checkpoint by phosphorylating p53 and CHK2 [139,146,147,148]. 

ATR, a 303 kDa PIKK protein, has been shown to associate with DNA lesions at ssDNA to dsDNA transitions, such as those that are present following dsDNA resection. ATR is also recruited to stalled replication forks during DNA replication where it plays a central role in the replication checkpoint [149]. Like ATM, ATR phosphorylates serine and threonine residues in SQ/TQ sequences [150,151]. Large scale proteomic analysis identified IR-induced ATM and ATR phosphorylation on the SQ/TQ consensus site for more than 700 proteins [135,152]. This number of proteins, and the broad range of different pathways affected, underscores the central role of ATM and ATR in the DDR. 

This central role of ATR demands tight control of its activity. For full activity, it needs to interact with specific protein complexes at the chromatin. In response to DSBs, ATM and the MRN complex recruit CtIP to DSBs where MRN and CtIP cooperatively resect the DSB [67,137,153,154]. The resulting ssDNA is coated and stabilized by replication protein A (RPA), which recruits the RAD17/RFC2-5 complex to ssDNA. The 9-1-1 (RAD9-RAD1-HUS1) complex is then loaded by Rad17/RFC2-5 complex and recruits the Topoisomerase II binding protein (TopBP1) to dsDNA-ssDNA junctions [155,156]. RPA recruits ATR together with the ATR interacting protein (ATRIP) to the DNA damage site where ATRIP interacts with TopBP1 to fully activate ATR [156,157] (see Figure 1). Whereas the ATM response to DSBs is very rapid and cell cycle independent, the ATR response requires CDK-dependent resection and is restricted to the S and G2 phases of the cell cycle [137].

#### 3.2.5. Cell Cycle Kinases CHK1 and CHK2 in Response to IR

The checkpoint kinases CHK1 and CHK2 are key effector proteins in regulating cell cycle arrest by phosphorylating CDC25 phosphatases, leading to CDC25 degradation and/or inhibition and thus to cell cycle arrest by CDK inactivation. The main activator of CHK1 in response to DNA damage is the ATR kinase, which phosphorylates CHK1 on S317 and S345 [137,158,159]. In response to DSBs, CHK1 can be activated indirectly by ATM via DSB resection and subsequent ATR recruitment. In addition, ATM may also directly target and activate CHK1 [160]. Despite the fact that CHK2 is structurally unrelated to CHK1, the checkpoint kinases have some degree of functional overlap. CHK2 activation requires phosphorylation on T68, mediated by ATM in response to DSBs [161]. Studies have suggested a role for CHK2 in the control of the G2 checkpoint [162,163]. However, the importance of CHK2 in the G2/M checkpoint is still under debate since experiments in CHK2-deficient mice and cells indicate that it is important for p53-dependent apoptosis but dispensable for G2/M arrest after damage [164,165]. Moreover, CHK2, unlike CHK1, is redundant for the degradation of CDC25A in tissue culture cells [166]. It is, however, possible that resection deficient cells, or other specialized situations, will display a strong dependency for CHK2 in checkpoint control due to increased ATM activity. A model has been proposed where the two checkpoint kinases CHK1 and CHK2 work in parallel in response to DSBs to orchestrate the checkpoint response throughout the cell cycle. CHK2 is activated by ATM in a cell cycle independent manner. During the S and G2 phase, CHK1 is activated by ATR following ATM and MRN-mediated resection of the DSB [167] (See Figure 1).

#### 3.2.6. The CDC25 Family as CHK1 and CHK2 Targets

The CDC25 phosphatases activate Cyclin-CDK complexes by removing inhibitory phosphorylations from CDKs. In response to DNA damage, CHK1 and CHK2 phosphorylate CDC25C to create a binding site for the 14-3-3 protein. Subsequently, the 14-3-3 protein sequesters CDC25C away from its substrates, notably CDK1 [32,168,169,170]. For the IR-induced G2/M checkpoint, the ATR-CHK1 pathway has been shown to be the main regulator of CDK1-Cyclin B complex activity, via control of the CDC25A protein level [171,172]. CHK1 phosphorylation of CDC25A leads to its polyubiquitinylation and degradation by SCF-βTRCP during S and G2 phases following DNA damage [173,174]. CDC25B- and C-deficient cells on the other hand, are viable and have a normal G2/M checkpoint [175,176]. CDC25B and C may thus function as a backup for CDC25A.

A further layer of control of CDC25A degradation following IR is achieved by the kinase NEK11. It was identified as a member of the CHK1-CDC25A pathway enforcing the G2/M checkpoint. CHK1 phosphorylates and activates NEK11, which in turn directly phosphorylates CDC25A, thereby promoting the SCFβTRCP-dependent degradation of CDC25A as presented [177]. Thus, CHK1, together with NEK11, phosphorylates CDC25A in response to DSBs, initiating its degradation. Subsequently the Cyclin B-CDK1 complex maintains its inhibitory phosphorylation and arrests the cell in the G2 phase (See Figure 1).

## 4. The Regulation of the IR-Induced G2/M Checkpoint

**Figure 2:**
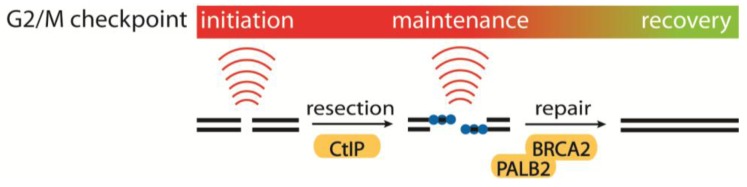
Initiation, maintenance and recovery of the G2/M checkpoint.DNA double strand breaks (DSBs) initiate the G2/M checkpoint in an ATM-dependent manner thereby blocking CDK1 activity. In the course of DSB repair, CtIP facilitates DNA end resection, maintaining the G2/M checkpoint. The checkpoint is further maintained by BRCA2, PALB2, and BRCA1 (not shown) during homologous recombination repair. Upon repair of the damaged DNA, CDK1 is reactivated, promoting recovery from the checkpoint.

The IR-induced G2/M checkpoint can be understood as a process in three phases: initiation, maintenance, and recovery. Each of these phases is regulated by a number of pathways with varying importance depending on the status of the on-going repair and the presence of repair intermediates. This supports the original concept of checkpoints: to allow time for repair. Interestingly, several proteins of the slow component of DNA DSB repair, HR, have been shown to be critical for either checkpoint maintenance, or the prevention of premature checkpoint recovery [3,5,6] (see Figure 2).

### 4.1. Initiation, Maintenance and Recovery of the G2/M Checkpoint

DNA DSBs formed after exposure to IR can give rise to a variety of chemically heterogeneous DSB ends, including those with modified terminal nucleotides or even protein-DNA adducts, often referred to as ‘dirty’ ends. These ends require processing by nucleases or other DNA modifying enzymes to enable repair by HR or NHEJ [58]. As presented, the DNA DSB is detected by the MRN complex, which tethers the DNA ends and potentially recruits ATM to the DSB. The DSB ends formed after the initial resection have 5’-P and 3’-OH groups suitable for ligation or priming DNA synthesis. It can be speculated, that this structure is sufficient for ATM activation, leading to checkpoint initiation as well as initiation of DNA end resection via CtIP activation. AT patient cells have been used to verify the strong dependency of ATM for the initial checkpoint [123]. In addition, it is possible that short ssDNA junctions generated at the newly formed DSBs activate the ATR pathway very rapidly after break formation [178]. These junctions may provide a loading site for the 9-1-1 complex during S and G2 phases, thereby recruiting TopBP1 to activate ATR [157,179]. Moreover, a number of studies have suggested that the MRN complex is also required for this initial activation. [73,180]. Further studies are needed to fully establish a molecular understanding of how the initial checkpoint is activated and controlled. It is likely that this will require an improved understanding of how ATM is activated following IR. 

Following the initiation of the G2/M checkpoint, the DNA end resection leads to a switch from ATM to ATR dependency for the checkpoint control [181]. This switch is dependent on the ssDNA tracts formed by DNA end resection. For this process CtIP is critical, and in a recent publication from our lab, this role of CtIP was linked to its role in maintaining the G2/M checkpoint [5]. Importantly, initiation of the G2/M checkpoint does not require extensive DNA end resection since CtIP-depleted cells were proficient in the initial checkpoint activation. However, in the absence of CtIP cells enter mitosis much earlier than normal, and the checkpoint abrogation occurs during the period when the resected DNA would normally be present. This temporal correlation indicates that there is a relation between the role of CtIP in regulating the G2/M checkpoint and its role in DNA end resection. In line with this, cells expressing CtIP mutants that are unable to promote DNA end resection were also deficient in checkpoint maintenance [5,67,182]. Another recent study has suggested that CtIP depleted cells repair some DNA lesions more rapidly after IR due to an increase in the activity of NHEJ in the absence of functional HR repair [63]. This could indicate that CtIP-depleted cells are entering mitosis at an earlier time point than wild type cells after IR, because the lesions have been repaired. However, it has been shown that CtIP depleted cells progress into mitosis with persisting DNA damage [5]. Furthermore, it is possible that an additional contribution to checkpoint maintenance could come from the involvement of CtIP in transcriptional control of key cell cycle regulators [183,184,185]. Further studies are therefore needed to fully establish how CtIP, as well as other HR factors, control G2/M checkpoint maintenance via DNA end resection, transcriptional control, and potentially even other biological processes.

Another layer of control for maintenance of the G2/M checkpoint originates from the p53 pathway. p53 is extensively modified by phosphorylation and acetylation among others. Upstream regulators include ATM, ATR, DNA-PK, CHK1 and CHK2. The post translational modifications (PTMs) of p53 reduce the affinity of p53 for its negative regulator Mdm2 releasing p53 to initiate transcription of its targets including the CDK inhibitor p21 [186]. Through transcriptional induction of p21, p53 suppresses CDK activity, which allows activation of the pRb tumor-suppressor. Active pRb reduces E2F activity, thereby lowering the level of E2F transcriptional targets such as the APC inhibitor EMI1. This down-regulation promotes premature APC activation in G2 phase cells and results in the degradation of key mitotic proteins such as Cyclin A and Cyclin B [187]. Moreover, p53 has also been suggested to be capable of controlling the G2/M checkpoint independently of p21 through transcriptional repression of mitotic inducers, including Cyclin-B, CDC25B and Plk1 [3].

**Figure 3:**
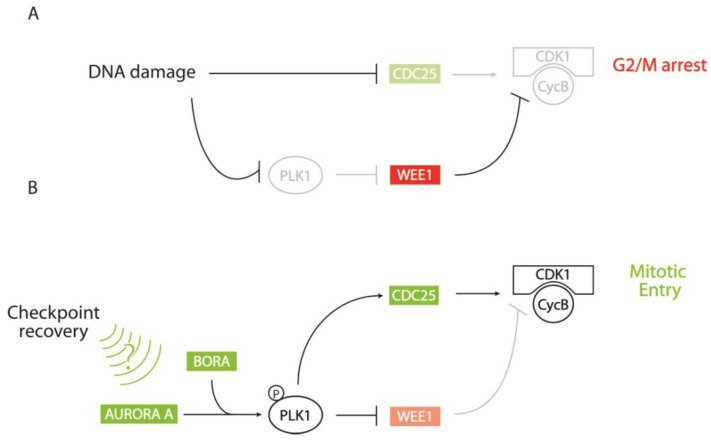
Plk1-dependent recovery from DNA damage induced cell cycle arrest. (A) In response to DNA damage, cells arrest in G2 due to inhibition and degradation of CDK1 activators (like CDC25) and activation of CDK1 inhibitors (like WEE1). (B) Upon DNA damage repair, PLK1 is phosphorylated by AURORA A and BORA in a yet unknown manner. The activated PLK1 in turn phosphorylates the CDK1 inhibitor WEE1, targeting it for degradation, and the CDK1 activator CDC25 to promote its translocation to the nucleus.

Checkpoint recovery is an active process to resume the cell cycle upon DNA damage repair completion. G2/M checkpoint recovery can be regarded as a special form of mitotic entry with alterations in the mitotic entry network, such as CDC25A degradation. Subsequently, AURORA A, BORA, and PLK1 are required for checkpoint recovery, whereas they are largely redundant for mitotic entry in unperturbed cells [38,39,50,188,189]. In order to facilitate checkpoint recovery, PLK1 becomes activated by AURORA A/BORA-mediated phosphorylation on T210 within its activation loop in a yet unknown mechanism [38,39]. PLK1 modulates regulators of CDK1 to finally promote mitotic entry. (See Figure 3) Firstly, CDK1 phosphorylates its inhibitory kinase WEE1, targeting it for a second phosphorylation by PLK1. Dual phosphorylated WEE1 can be targeted for polyubiquitinylation by SCF-βTRCP for proteasomal degradation, thus facilitating mitotic entry [190,191]. Secondly, another CDK1-inhibiting kinase, MYT1, is also phosphorylated by PLK1, resulting in an inhibition of its kinase activity [29]. Finally, PLK1 can phosphorylate the CDK1 activator CDC25C to promote its accumulation in the nucleus [192,193]. Whether PLK1 also targets CDC25A and B is currently unclear. Interestingly, the SCF-βTRCP complex also targets CDC25A for degradation during G2/M checkpoint initiation, implying that two opposing processes (checkpoint initiation and recovery) are under control of the same complex. This points out the importance of upstream phosphorylation of the SCF-βTRCP complex for its substrate specificity [194]. Moreover, PLK1 may be inhibited during checkpoint initiation due to the protein phosphatase 2 A (PP2A) suppressing the activating T210 phosphorylation [195,196,197].

Emerging data suggest intimate connections between homologous recombination pathways and checkpoint pathways. Through unbiased siRNA screening, BRCA2 and PALB2 have been identified as key regulators of G2 checkpoint maintenance and, importantly, their function seems to be independent of homologous recombination, as RAD51 depletion does not affect G2 checkpoint control [4,6]. BRCA2 and PALB2 were shown to be important for preventing premature activation of PLK1. It now remains to be determined how BRCA2 and PALB2 mediate control of the PLK1 pathway and how it is turned off to allow cell-cycle progression. An exciting aspect is clearly how this new checkpoint function might contribute to BRCA2 and PALB2 tumor-suppressor functions. 

## 5. Perspectives

A detailed understanding of the DDR is of great importance for our understanding of cancer development and cancer therapy. First, it is known that mutations in DDR genes can lead to cancer predisposition [1,2,3,198]. It is thus important to elucidate the functional roles of these factors. Second, in the context of cancer treatment, an in-depth understanding of the response to various DNA damage lesions, and how these are deregulated in cancer cells, can pave the way for identifying the Achilles’ heel of these cancer cells [199]. The technical progress of the last years has made it possible to create and handle siRNA libraries to target genes on a high‐throughput level. For instance, the homologous recombination protein BRCA2 and the previously uncharacterized protein RHINO were identified as G2/M checkpoint regulators in siRNA screens [4,6]. These approaches can help identify new targets for cancer therapy.

Ionizing radiation (IR) is frequently used in the clinic as a cancer treatment where the tumor cells are mainly killed due to massive amounts of DNA double strand breaks. IR therapy is normally accompanied by severe side effects and is often not fully effective due to the acquired resistance of tumor cells. The obtained resistance can be caused by activated cell cycle checkpoints. Thus, G2/M checkpoint regulators like CtIP, BRCA2 and PALB2 are potential targets for cancer therapy. Moreover, knowing the functional status of checkpoint regulators in a tumor can be beneficial for a rational treatment strategy. For example, the increased radiation sensitivity observed in BRCA2‐defective tumors [200], which has previously been attributed to homologous recombination repair defects, could thus partly result from G2/M checkpoint abrogation in these cells.

Inhibiting the G2/M checkpoint in combination with IR can potentially target a broad range of tumors. More than 50 % of human tumors are deficient of the tumor suppressor protein p53 [201]. Cells lacking the p53‐dependent G1 checkpoint may be more dependent on the G2/M checkpoint for repair of DNA damage [202]. As presented, p53 has been reported to be required for G2/M checkpoint maintenance. However, these studies have also shown that p53-independent pathways are sufficient to induce G2 arrest, and that p53 probably plays a role in ensuring the long-term duration of the arrest [17,203]. In line with this, a recent study suggests a switch from p21-dependent to CHK1-dependent G2 arrest in p53 deficient cells [165]. Further studies are needed to elucidate the interplay between the different pathways governing the IR-induced G2/M checkpoint. These findings could pave the way for a rational combined treatment of ionizing radiation in with a G2/M checkpoint inhibitor to specifically target and kill p53‐deficient tumor cells. 
