# Correction: Kousholt, A.N. *et al*. Pathways for Genome Integrity in G2 Phase of the Cell Cycle. *Biomolecules* 2012, *2*, 579-607

**DOI:** 10.3390/biom3010072

**Published:** 2013-01-15

**Authors:** Arne Nedergaard Kousholt, Tobias Menzel, Claus Storgaard Sørensen

**Affiliations:** Biotech Research and Innovation Centre, University of Copenhagen, Ole Maaløes Vej 5, DK-2200 Copenhagen N, Denmark, E-Mails: ank@bric.ku.dk (A.N.K.); TME@bric.ku.dk (T.M.)

We have discovered an error in our paper published in Biomolecules [1], in Figure 1 on page 589. The protein names ATR and ATRIP have been swapped. A corrected version of the Figure 1 is provided below.

**Figure 1:**
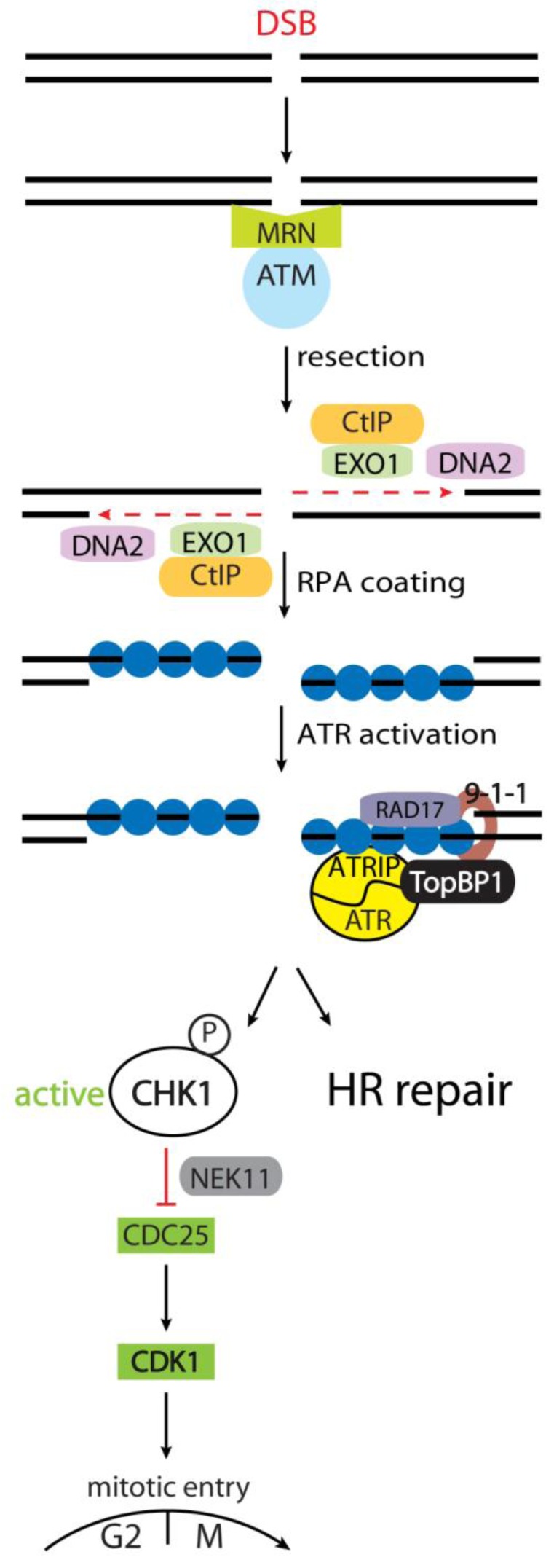
Activation of the checkpoint regulator (CHK1) in response to DNA Double Strand Breaks (DSBs). The MRN complex detects DSBs and recruits Ataxia Telangiectasia Mutated (ATM) to initiate checkpoint signaling. During the S and G2 phases of the cell cycle, dsDNA resection can be performed by the nucleases DNA2 and EXO1, together with CtIP. The resulting ssDNA is coated by RPA, and is prepared for HR repair. Furthermore, ssDNA-coated by RPA recruits the ATR/ ATR interacting protein (ATRIP) complex as well as TopBP1 via the 9-1-1 complex, enabling full ATR activity. ATR then activates a subset of targets, including CHK1. CHK1 can phosphorylate CDC25A in response to DSBs, targeting CDC25A for proteasomal degradation. Since CDC25A is required for CDK1 activity to facilitate mitotic entry, cells arrest in the G2 phase upon CDC25A degradation.
